# Rapid Weight Loss Across Combat Sports and the Relationships Between Methods and Magnitude

**DOI:** 10.1155/tsm2/2946317

**Published:** 2025-03-26

**Authors:** Oliver R. Barley, Craig A. Harms

**Affiliations:** ^1^School of Medical and Health Sciences, Edith Cowan University, Joondalup, Western Australia, Australia; ^2^School of Arts and Humanities, Psychology and Criminology, Edith Cowan University, Joondalup, Western Australia, Australia

## Abstract

This study examined rapid weight loss (RWL) habits across combat sports (CS) and how common usage of different methods was related to the magnitude of RWL. Competitors (*N* = 256) from CS including boxing, Brazilian jiu-jitsu, muay thai/kickboxing (MT/KB), wrestling, mixed martial arts (MMA), judo, taekwondo, and karate participated in the study. Athletes completed an online survey which included questions on their regular weight loss habits, including magnitudes of weight loss over different time periods and types of methods utilised. Athletes typically started losing weight in their early 20s and engaged in RWL on average three times a year. Magnitudes of weight loss were greater in MT/KB and MMA than other cCS examined (*d* between 0.63 and 1.54). Wrestlers demonstrated higher prevalence of skipping meals or fasting than other CS (*d* between 0.7 and 1.29). Athletes at higher competitive levels engaged in larger magnitudes of RWL (*d* between 0.49 and 0.57). The usage of methods of body fluid manipulation such as fluid restriction, water loading, and sauna were associated with greater amounts of weight loss within 2 weeks and 24 h of weighing in (*d* between 0.36 and 0.45). Findings indicate that larger weight cuts are linked to strategies involving higher risk, such as sauna, and may be more common among athletes who begin RWL practices at a younger age. Use of certain high-risk methods were associated with increased likelihood of disordered eating behaviours. To reduce reliance on these practices, practitioners and regulators should emphasise the use of smaller weight cuts and to begin losing weight further out from competition.

## 1. Introduction

It is common practice for combat sports (CS) athletes to intentionally and rapidly lose significant amounts of weight prior to being weighed before a fight [1–3] in order to record a weight for a weight class lower than their typical noncompetition weight and gain a competitive advantage. Such precompetition RWL is commonly followed by rapid weight gain after weigh-in, which is part of an overall weight cycling process in CS. Very high prevalence rates of RWL in CS have been reported by most (> 80%) of the participants [1, 4, 5] to more moderate (∼60%) levels [6, 7]. RWL can include energy intake restriction or expenditure that might be viewed as healthy (e.g., gradual dieting and exercising more than normal) or unhealthy (e.g., skipping one or two meals per day as well as fasting); unhealthy body fluid management strategies (e.g., restricting fluid ingestion or excessive fluid ingestion); unhealthy practices for removal of body fluid (e.g., heated wrestling, plastic suits, saunas, and spitting); and pseudo extreme (vomiting) or inappropriate use of medical practices (e.g., use of laxatives, diet pills, diuretics, and enemas) [1]. Despite the commonality of RWL in CS, the influence of RWL strategies and physical and competitive performance in CS is somewhat unclear [8], with research reporting greater magnitudes of weight loss and regain to improve [9–12], have no influence on [12–14], or impair competitive success [15]. Despite these inconclusive findings, the practice of RWL continues to persist; thus, a fuller examination of RWL is needed to fully understand the persistence of RWL.

While aspects of precompetition weight loss such as the typical and most weight lost before a competition, typical weight lost 2 weeks as well as 24 h before a competition, and the weight typically regained after weigh-in for a competition have been investigated by previous research [1, 16, 17], there is a paucity of data examining the surrounding CS-context of habits in rapid weight loss (RWL). As noted by Matthews et al. [18], their systematic review, and while acknowledging that data are not available for all CSs, the amount of body mass lost prior to competition can differentiate across different CSs, with MMA athletes tending to lose more body mass than wrestlers. Differences for precompetition weight loss history according to CS and level of competition may be evident given that Amatori et al. [4] found that the most severe cluster of users of RWL was related to the level that the athletes participated (i.e., higher for professional and high-level athletes). Differences in usage of RWL according to the CS and the level that the participants competed at may provide evidence of CS sport-specific cultural difference regarding RWL usage before competition.

The link between the use of RWL methods and the athlete's history of weight loss magnitude has not been well explored. Historical associations between the various RWL methods and weight lost prior to competition may shape athlete expectations about the effectiveness of RWL behaviours which in turn may impact on the use of the RWL methods in the future. For example, if a certain RWL method is, in the mind of the athlete, associated with a large amount of weight lost in the 2 weeks prior to competition for a CS athlete, and this athlete felt that losing a large amount of weight 2 weeks prior to a competition was a desirable outcome, then there may be a greater chance that an athlete would use this RWL method. Better understanding this may identify which methods are more problematic for athletes engaging in larger RWL. Differences in these associations between CS sports and level of competition may further indicate CS-specific cultural differences in RWL.

The present study had two broad aims. The first aim was to report on the CS-context (e.g., level of competition and type of CS) for a broader profile of weight loss history for CS that is typically examined in CS research including various measures of weight lost prior to competition (e.g., most weight lost before a competition as well as typical weight lost in the 2 weeks and 24 h before weigh-in) as well as the age that the CS athletes began losing weight for competitions and the recent frequency of weight loss before a competition (e.g., in the last 2 years). The second aim was to report on the associations between RWL and this broader profile of weight loss history, with differences according to the CS context also examined.

## 2. Method

### 2.1. Participants

All participants were at least 18 years old and registered in CS organisations and/or were active members of CS gyms. Although 298 persons agreed to participate in a larger study, 256 participants consented to this study on the weight loss patterns in CS. Participants who were not at least 18 years old or had not competed within the previous 12 months were excluded from the study. The average age of these 256 participants was 28.42 years (± 9.5), most of the participants were male (85.9%), and most lived in the United States of America (48.7%), Australia (14.4%), the United Kingdom (12.1%), or Canada (5.4%). The participants described themselves as being at about the mid-stage of their career (*M* = 45.71, ± 32.74, on a visual analog scale ranging from 1 to 100, with higher scores indicating later career stage).

### 2.2. Measures

#### 2.2.1. Weight Loss Behaviours of CS Athletes

The participants completed a survey on weight loss behaviours adapted from a survey developed by Artioli et al. [16] for judo athletes. The participants answered questions about their current primary CS (boxing, Brazilian jiu-jitsu [BJJ], muay thai/kick boxing (MT/KB), wrestling, mixed martial arts [MMA], judo, taekwondo, and karate) and their current level of competition (amateur, regional/state, and elite) for this CS. As noted previously [19], the athletes who reported participation in taekwondo and karate were combined as a traditional striking sport (TSS) group because of smaller sample sizes recruited across both groups, which has been done in previous research [19]. While there are similarities between taekwondo and karate, they are still different sports, so the results should be interpreted with caution.

The participants were asked about aspects of their precompetition weight loss history before competition: if they had ever lost weight before a competition (yes or no); the age when they had begun weight loss prior to competition, and how often they had lost weight before a competition in the previous 2 years (5-point Likert scale: 1 = never, 2 = a few, 3 = sometimes, 4 = most; 5 = every time) as a measure of recency of RWL. The participants were questioned about several historical weight loss outcomes (in kilograms or pounds, with weight in pounds converted by the researchers to kilograms): amount of weight typically lost for a competition; most weight lost for a competition; weight typically lost in the 2 weeks as well as 24 h before the weigh-in; and, following the weigh-in, weight typically regained after weigh-in and prior to competition.

Next, participants were asked about their typical usage of the following precompetition weight loss methods: gradual dieting for more than 2 weeks; increased exercise (more than usual); skipping one or two meals per day; fasting (not eating all day); restricting fluid ingestion; excessive fluid ingestion (water loading); training in heated rooms; sauna; plastic/rubber suits or towel wrapping; using winter or plastic suits for whole day (without exercising); spitting; laxatives; diuretics; diet pills; and vomiting. Participants rated their typical usage of these methods on a 4-point Likert scale (1 = never, 2 = almost never, 3 = sometimes, and 4 = always).

### 2.3. Procedure

Participants were informed of the study procedures and consented to participate in the study by accepting the terms and conditions before data collection. All experimental procedures were approved by Edith Cowan University's Human Research Ethics Committee (Research Ethics Identification: 2019-00278-BARLEY). The participants completed the questionnaire in Qualtrics (Qualtrics August to December 2019, Qualtrics, Provo, Utah, USA), with the link to the survey e-mailed to the participants by CS gyms/organisations/message boards around the world who agree to send out the link to the survey. Where the relevant moderators approved, advertisements about the study were posted on several message boards where potential participants might visit.

### 2.4. Statistical Analysis

An initial inspection of the data resulted in re-coding of responses for several methods of RWL—plastic rubber suits or towel wrapping, wearing winter plastic suits for the whole day without exercising, spitting, laxatives, diuretics, diet pills, and vomiting—as being “a history of use” or “never used” because the responses were essentially dichotomous, with most participants (between 89% and 52%) reporting that they had never used one of these methods of RWL. As such, these methods of RWL were categorised as “less common”. To distinguish them from the “less common” methods of RWL, the other methods of RWL—more exercise than normal, skipping one or two meals per day, fasting, restricting fluid ingestion, excessive fluid ingestion, training in heated rooms, and sauna use—were categorised as “common” methods of RWL.

For the precompetition weight loss history as well as common methods of RWL, preliminary analysis involved examining the data for univariate outliers prior to reporting of descriptive data (M and SD). Differences according to (three) levels of competition and (seven) primary sports were examined by one-way ANOVA, with Cohen's criteria (Cohen, 2013) used to describe effect size (*d*) where post-hoc tests identified differences between groups. Along with frequency data, differences for less-common methods of RWL for “a history of use” and “never used” according to the level of competition were examined using *χ*^2^ test for independence.

The examination of the relation between methods of RWL and precompetition weight loss history was examined using Pearson *r* correlation, with moderation effects (by multigroup analysis) according to level of competition also examined (Table 1), with Cohen's criteria (Cohen, 2013) used to describe effect size. For the less common methods of RWL, this relation was examined using one-way ANOVA, with Cohen's criteria (Cohen, 2013) used to describe effect size (*d*) where post-hoc tests identified differences between groups (Table 2). Analyses for one-way ANOVA, Pearson *r*, and *χ*^2^ test for independence were conducted in SPSS version 28. Multigroup analysis was conducted in Mplus Version 8.3 [20]. The value for alpha in all analyses was 0.05. See Appendix A for details of the analyses.

## 3. Results

### 3.1. Preliminary Analysis

All participants reported losing weight at some point in their career prior to a competition. Extreme values (i.e., univariate outliers) were found on at least one measure of precompetition weight loss history for 12 participants (4.6% of the total sample). These participants were mostly male and reported a variety of CS backgrounds; and just over half (seven) reported some history of using the less-common methods of RWL before competition (see Appendix A for details). The data of these 12 participants were retained for further analyses as the responses were considered by the first author, who is an experienced researcher in CS, to be believable.

### 3.2. Profile of RWL and Precompetition Weight Loss History (Table 3)

Before competing, the participants reported their typical usage of three commonly used methods of RWL—more exercise than usual, gradual dieting, and excessive fluid ingestion—as being between “sometimes” and “always;” and their typical usage of the other common methods of RWL as being between “almost never” and “‘sometimes.” Some usage of the less common methods of RWL was reported by between 12% and 48% of the participants. The participants began losing weight for a competition between their 20^th^ and 21^st^ birthdays, and they lost weight prior to a competition between three and four times in the previous 2 years. The participants reported the most weight for a competition was close to 8 kg; they typically lost just over 5 kg prior to a competition, between 3 and 4 kg in the 2 weeks before a competition, and between 1 and 2 2 kg in the 24 h before competition, and they typically regained between 2 and 3 kg after weigh in.

Some differences in these measures were found for the competition level. Compared to amateurs, elite athletes reported lower use of gradual dieting, being younger when losing weight before a competition (medium effects), and more frequent use of plastic suits and towel wrapping, winter or plastic suits without exercising (medium effects). Elite athletes reported greater typical weight loss in the 24 h prior to a competition compared to state/regional athletes (medium effect). State/regional athletes reported less frequent weight loss before a competition compared to both amateurs and elite athletes but greater typical weight loss before a competition compared to amateur athletes (medium effects). Amateur athletes reported less frequent use of spitting (medium effect).

More differences were noted for primary sport, with only differences compared to at least two sports noted here. Wrestlers report greater usage of skipping meals (compared to four CSs), fasting (compared to two CSs), training in heated rooms (compared to five CSs), and sauna usage (compared to two CSs). All effects were medium. Wrestlers began losing weight for competition at a younger age compared to three CSs (medium effects), and they reported greater weight loss in the 2 weeks prior to competition compared to four CSs (medium effects). TSS athletes had less use of restricting fluid ingestion compared to three CSs and less use of sauna compared to five CSs (medium effects). Judo athletes reported less frequent weight loss before a competition compared to three CSs (medium effects). MT/KB athletes reported greater use of excessive fluid ingestion compared to four CSs. MT/KB and MMA athletes reported a larger value for typical weight loss and weight lost in the 2 weeks before competing to BJJ and Judo (medium effects). MT/KB also reported greater weight lost in the 2 weeks prior to competition compared to TSS athletes. MMA and MT/KB reported losing more weight in the 24 h before competition compared to four and three CSs, respectively. MMA and MT/KB reported gaining more weight after weight in compared to four and five CSs, respectively.

### 3.3. Association Between Common Methods of RWL and Precompetition Weight Loss History (Table 1)

Greater use of gradual dieting was associated with being older when beginning weight loss prior to competitions and greater values for one of the five weight-loss outcomes (small effects). Greater use of skipping meals was associated with being younger when beginning losing weight before a competition and greater values for two of the five weight loss outcomes (small effects). More exercise than normal was associated with being younger when beginning weight loss prior to competitions and higher values for three of the five weight loss outcomes (small effects). Greater use of fasting was associated with being younger when beginning losing weight before a competition and greater values for all five weight loss outcomes (small effects).

Greater use of restricting fluid ingestion was associated with being younger when beginning losing weight before a competition (small effect), with this effect being stronger for state/regional athletes and elite athletes (medium effects); greater frequency of weight loss before a competition in the previous 2 years (small effect); and greater values for all five of the weight loss outcomes (small or medium effects). Greater use of excessive fluid ingestion was associated with greater frequency of weight loss before a competition in the previous 2 years (small effect), with this effect being stronger for amateurs (medium effect); and greater values for all five of the weight loss outcomes (small or medium effects).

Greater use of heated rooms was associated with being younger when beginning losing weight before a competition (small effect), with this effect being stronger for state/regional athletes (medium effect), and greater values for all five of the weight loss outcomes (small effects). Greater use of sauna was associated with greater frequency of weight loss before a competition in the previous 2 years (small effect) and greater values for all five of the weight loss outcomes (small or medium effects).

### 3.4. Relation Between Less Commonly Used RWL Methods and Precompetition Weight Loss History (Table 2)

For five of the seven methods, participants with some history of the less common methods of RWL began losing weight for competition at a younger age compared to nonusers (at least medium effects), with greater (large) effects noted for spitting, diet pills, and vomiting. Participants who had some history of using plastic/rubber suits or towel wrapping (small effect) or diuretics (small effect) reported a greater frequency of weight loss before a competition in the previous 2 years.

Participants with some history of the less common methods of RWL reported greater values on all weight loss outcomes compared to nonusers (small to medium effects). The strongest effects were observed for weight loss 2 weeks and 24 h prior to competition, particularly for use plastic/rubber suits or towel wrapping (large effects).

## 4. Discussion

This study investigated the links between precompetition RWL practices and the weight loss histories of CS athletes. By profiling the weight loss behaviours of these athletes, we aimed to identify specific weight loss methods of concern and the habits of people engaging in greater RWL. This research is crucial for understanding how weight management practices may vary across different levels of competition and types of CS, impacting athlete health and performance.

Within this study, the participants reported typically beginning weight loss in their early 20s; they lost weight for a competition three times a year over the previous 2 years; their greatest weight loss was about 50% greater than their typical weight loss reduction prior to a competition; as a proportion of their typical weight loss for a competition, they lost 70% of their weight loss in the 2 weeks before a competition and 32% of the weight loss in the 24 h before competing; and they regained 48% of their typical weight loss prior to competition after weigh-in. These results are concerning as they outline that while there is high use of gradual dieting and increased exercise, large portions of athletes are doing significant portions of their weight cuts later in their preparatory phase and would not match healthy weight loss recommendations [21].

While CS all exist under the same umbrella, we found important differences between sports and levels of competition for different weight loss magnitudes prior to competition [2]. We observed that MT/KB and MMA generally lost more weight for competitions that most other CS included, especially within 24 h of the weigh-in (Table 3). With MMA, this is unsurprising and aligns with previous research [1, 3]; however, the larger RWL observed in MT/KB is somewhat surprising as previous research has reported it being more similar to boxing [1]. It is possible that with the growth of higher level MT/KB such as in One Championship within recent years, the sports have moved closer to the weight loss habits observed in MMA. Unlike Judo and BJJ, MMA and MT/KB typically host weigh-ins the day before the bout, allowing ∼24 h recovery, as opposed to the same-day weigh-ins in the former, which may enable larger weight cuts. Though part of the grappling-exclusive sports, wrestling led in weight loss magnitude (Table 3), which aligns with previous research [1, 17]. Though it is unclear why wrestling has larger weight loss magnitude than other grappling sports with same-day weigh ins, the differences may be indicative of a broader cultural issue within the sport [1, 2, 22]. Overall, these results indicate that the CS sports of MT/KB, MMA, and wrestling in this study had more severe RWL culture which should likely be the focus of future weight loss research and potential regulatory interventions. Regarding competitive level, we found that elite athletes reported competing more often as well as losing more weight within 24 h of the weigh-in than lower competitive levels (Table 3). While it is concerning, it stands to reason that elite athletes are willing to take greater risks in attempts to gain a competitive advantage. This outlines a potential risk of a RWL culture with higher level athletes which may pose serious risks to their health and well-being.

There is a large range of methods an athlete can use for RWL, with some being healthier and others posing a greater risk to athlete health. Interestingly, within the present study, we observed no differences between CS or competitive levels when it comes to the use of healthier methods of weight loss (i.e., gradual dieting and more exercise than usual). This lack of difference is similar to findings from previous research showing the prevalence of these methods to be high across all CS assessed [1, 6]. However, other findings indicated CS-context differences for precompetition RWL.

Wrestlers within the present study reported significantly higher usage of skipping meals or fasting entirely (Table 3), which may put wrestlers at higher risk of eating disorders [23, 24]. While there is minimal research in disordered eating in CS [24], further research is required in the sport of wrestling. The sauna, which is one of the most controversial of the RWL techniques, was also observed to be higher in wrestling than other grappling sports, which further supports a greater RWL culture within the sport (Table 3). Conversely, TSS reported less reliance on saunas for making weight, which combined with lower magnitude of weight loss indicates a potentially less severe RWL culture. While previous research has reported significantly higher usage of saunas in MMA [1], we found their rate to be high but not significantly greater than other sports. It is unclear if this is because of other sports introducing greater sauna reliance or MMA reducing their own. As athletes reach higher competitive levels, it is plausible that they would take greater risks to make weight, hoping for potentially larger competitive advantages. Within this study, we observed that elite athletes were more likely to have engaged in the use of plastic/rubber suits or towel wrapping, as well as the use of winter or plastic suits for an entire day without exercising (Table 3). Such results align with previous research showing higher level athletes using riskier weight loss strategies to make weight [4] and outline that regulatory body may need to divert greater attention to higher level competitors when attempting to manage RWL practices.

Within the present study, there were some concerning results when looking at how weight loss magnitude was related to the methods utilised. Indeed, larger weight loss within 2 weeks of the competition, and within 24 h before the weigh-in, was associated with increased use of fluid restriction, water loading, and sauna usage (Table 1). Athletes who utilised spitting or plastic/rubber suits or towel wrapping began cutting weight younger, cut weight more frequently, lost more weight within 2 weeks and 24 h of the weigh-in that those that did not. Taken together, these results indicate that when athletes are attempting larger weight cuts, they are more likely to resort to higher risk weight loss methods, which align to previous research conducting cluster analysis on boxers' weight loss habits [4]. The usage of strategies that significantly alter whole body water, and thus blood viscosity, has been a significant source of concern regarding athlete safety [3, 25].

Of the less commonly used methods in precompetition RWL, the participants that reported some use of laxatives, diet pills, and vomiting lost more weight within 2 weeks of the competition but not 24 h before competition (Table 2), which may indicate that while such methods are not ideal from a health perspective, they are not methods of choice for athletes trying to lose large amounts of weight very close to weighing in. However, the usage of diet pills and vomiting may indicate a potential for an eating disorder problem within CS [24, 26]. Surprisingly, there is minimal research of the prevalence of eating disorders in CS which should be addressed in the future. Regarding competitive levels, elite athletes were more likely to utilise restriction of fluid ingestion at a younger age than other competitive groups. Examining our results more broadly, there were concerning trends around those that began cutting weight younger in life utilising higher risk strategies (Tables 1 and 2). When attempting to develop strategies to change the culture of RWL within CS, it may be important to educate and regulate younger athletes to prevent them from developing higher risk habits as they age.

While the present study had several strengths, there are some limitations that need to be considered when interpreting the findings. The findings were based on self-reported data from CS gyms and online forums which means the accuracy may be affected by the athlete's memory and motivation, while a sufficiently large sample was collected in an attempt to mitigate this factor; the results need to be interpreted with caution. Even though the overall sample was large, the split among the different CS/competitive levels was uneven, and thus, some of the sports had smaller samples. As a result, we were unable to analyse some findings according to the athlete's primary sport/competitive level, which means there may be more depth to this topic that we were unable to explore. One feature of the findings was that several extreme values were observed for some participants, with most of these extreme values associated with weight loss outcomes. There was no clear way of describing their backgrounds regarding primary CS or level of competition. It is clear that there are certain clusters within CS that are doing more extreme cuts [4], and it is possible that this smaller population are the ones that require focussing on for regulatory purposes. Future research should aim to examine such athletes in more detail. Finally, it is important to note that while survey data presented within the present study are useful for informing broader decision-making, it is not necessarily representative of the individual, so careful consideration must be made when working with specific athletes or trying to fairly design policies to apply across large populations.

## 5. Conclusion

While the purpose of this study was to examine the habits of RWL within CS to further examine the differences between CS and how different weight loss practices may influence weight loss magnitude, such findings can be useful for the decision-making of regulators and researchers alike. One key finding of the present study was that larger weight cuts were associated with increased use of fluid manipulation (and potentially higher risk) strategies for making weight. We also noted that in many cases, the use of higher risk weight loss methods was associated with starting RWL at a younger age, with certain RWL methods putting CS athletes at risk of developing patterns of disordered eating. Future research would benefit from investigating differing strategies to minimise the amount of weight lost for competition. Practitioners and regulators need to be aware that one of the largest risk factors associated with RWL is the amount of weight lost; if athletes can be encouraged to lose less weight, they are less likely to resort to higher risk practices. While the present study provides greater context on weight loss habits in CS, it is important for future research to work collaboratively with practitioners to determine effective methods to manage higher risk weight loss habits, especially within the more concerning populations such as MMA, MT/KB, and youth athletes.

## Figures and Tables

**Table 1:** Statistically significant correlations between common methods of RWL before a competition and precompetition weight loss history.

	Age began losing weight for competitions (years)	Frequency of weight loss before a competition in the last 2 years (Likert 1–5)	Typical weight loss before a competition (kg)	Most weight loss before a competition (kg)	Typical weight loss in the 2 weeks prior to a competition (kg)	Typical weight loss in the 24 h prior to a competition (kg)	Weight gain after weigh-in for a competition (kg)
*Typical usage of….(Likert 1–4)*							
More exercise than usual	−0.14		0.18	0.16	0.16	—	
Gradual dieting	0.13		0.16				
Skipping one or two meals	−0.14			0.14	0.19		
Fasting	−0.20		0.12	0.23	0.21	0.14	0.17
Restricting fluid ingestion	−0.25 **(S/R = **−**0.32; El = **−**0.41)**	0.19	0.18	0.22	**0.38**	**0.40**	0.28
Excessive fluid ingestion		0.25 (**Am = 0.41** El = 0.28)	0.29	0.15	**0.36**	**0.47**	**0.45**
Training in heated rooms	−0.28 **(S/R = **−**0.42)**	(0.23 Am)	0.19	0.24	0.28	0.23	0.15
Sauna		0.13	0.29	0.22	**0.38**	**0.45**	**0.41**

*Note:* Findings in bold were medium effects. All findings appear in Appendices E and F.

Abbreviations: Am = amateurs, El = elite, S/R = state or regional.

**Table 2:** Statistically significant findings for precompetition weight loss history for less common methods of weight loss before a competition.

Some history of use compared to non-use of…	Age began losing weight for competitions (years) M & SD/*d*	Frequency of weight loss before a competition in the last 2 years (Likert 1–5) M & SD/*d*	Typical weight loss before a competition (kg) M & SD/*d*	Most weight loss before a competition (kg) M & SD/*d*	Typical weight loss in the 2 weeks prior to a competition (kg) M & SD/*d*	Typical weight loss in the 24 h prior to a competition (kg) M & SD/*d*	Weight gain after weigh-in for a competition (kg) M & SD/*d*
Plastic/rubber suits or towel wrapping	**Began younger**	**More frequent**	**Lost more**	**Lost more**	**Lost more**	**Lost more**	**Gained more**
	18.71 (5.21) < 21.93 (7.11)/0.52	4.10 (1.30) > 3.38 (1.37)/0.54	5.98 (2.72) > 4.39 (2.87)/0.57	9.11 (4.30) > 6.75 (4.43)/0.54	4.54 (2.16) > 2.80 (1.66)/0.90	2.26 (1.65) > 1.07 (1.09)/0.85	3.12 (2.29) > 1.80 (1.68)/0.66

Use of winter or plastic suits for whole day (without exercising)	**Began younger**		Lost more	**Lost more**	**Lost more**	Lost more	Gained more
	18.14 (5.34) < 21.21 (6.60)/0.54		5.83 (2.79) > 4.91 (2.91)/0.33	9.61 (4.90) > 7.26 (4.21)/0.50	4.51 (2.17) > 3.32 (1.99)/0.56	2.16 (1.61) > 1.46 (1.43)/0.45	2.92 (2.14) > 2.31 (2.09)/0.29

Spitting	Began younger		Lost more	**Lost more**	**Lost more**	**Lost more**	Gained more
	16.85 (5.25) < 22.12 (6.29)/0.95		5.81 (2.75) > 4.84 (2.93)/0.35	9.45 (4.27) > 7.12 (4.45)/0.54	4.68 (2.25) > 3.12 (1.82)/0.74	2.25 (1.58) > 1.34 (1.38)/0.60	3.11 (2.02) > 2.16 (2.26)/0.45

Laxatives			Lost more	**Lost more**	**Lost more**	Lost more	Gained more
			6.28 (2.99) > 4.89 (2.82)/0.47	10.14 (4.84) > 7.35 (4.27)/0.59	4.67 (2.45) > 3.39 (1.93)/0.54	2.26 (1.74)/1.50 (1.41)/0.45	3.30 (2.57) > 2.26 (1.94)/0.42

Diuretics		More frequent	Lost more	Lost more	Lost more	Lost more	Gained more
		4.10 (1.31) > 3.64 (1.39)/0.35	6.36 (3.39) > 4.78 (2.71)/0.48	9.68 (4.81) > 7.48 (4.36)/0.47	4.53 (2.30) > 3.43 (2.00)/0.49	2.18 (1.63) > 1.52 (1.45)/0.41	3.19 (2.51) > 2.31 (1.98)/0.36

Diet Pills	**Began younger**		Lost more	**Lost more**	**Lost more**	Lost more	
	16.71 (5.11) < 20.93 (6.47)/0.80		6.41 (3.72) > 4.97 (2.71)/0.40	10.47 (4.51) > 7.50 (4.40)/0.66	4.81 (2.10) > 3.46 (1.98)/0.65	1.55 1.42 2.25 1.89/0.38	

Vomiting	**Began younger**			**Lost more**	**Lost more**	Lost more	Gained more
	15.63 (4.30) < 21.00 (6.44)/1.17			10.14 (3.74) > 7.60 (4.54)/0.66	4.94 (2.52) > 3.45 (1.98)/0.61	2.52 (2.11) > 1.53 (1.37)/0.49	3.40 (2.71) > 2.35 (2.00)/0.40

*Note:* Findings in bold were either medium or large effects. All findings appear in Appendices G, H, and J.

**Table 3:** Profile of precompetition RWL and precompetition weight loss history.

	Descriptive statistics		Any statistically significant group differences by level of competition or primary sport (*d*)
	M	SD	
More exercise than usual (Likert 1–4)	3.49	0.86	
Gradual dieting (Likert 1–4)	3.53	0.80	El < Am/0.43
Skipping one or two meals (Likert 1–4)	2.84	1.06	Wr > boxing (1.01), BJJ (1.09), TSS (1.18), & MT/KB (1.29). judo > MT/KB (0.70)
Fasting (Likert 1–4)	2.54	1.23	Wr > boxing (0.75) & BJJ (0.79)
Restricting fluid ingestion (Likert 1–4)	2.83	1.22	TSS < judo (0.79), MT/KB (1.06), & Wr (1.33).
Excessive fluid ingestion (Likert 1–4)	3.13	1.29	MT/KB > TSS (0.93), judo (0.96), & Wr (1.08) MMA > Wr (0.81)
Training in heated rooms (Likert 1–4)	2.42	1.26	Wr > MMA (0.97), boxing (1.07), MT/KB (1.19), BJJ (1.45) & TSS (1.96). judo > TSS (0.91)
Sauna (Likert 1–4)	2.48	1.22	Wr > BJJ (0.80) & TSS (2.10). TSS < BJJ (0.93), boxing (1.19), MT/KB (1.48), MMA (1.63), & Wr (2.10)

	**Not used**	**History of use**	

Plastic/rubber suits or towel wrapping	132	124	El: less likely not to have used and more likely to have a history of use
Use of winter or plastic suits for whole day (without exercising)	180	65	Am: more likely to have not used and less likely to have a history of use El: less likely not to have used and more likely to have a history of use
Spitting	171	85	Am: more likely to have not used and less likely to have a history of use
Laxatives	206	50	
Diuretics	208	48	
Diet pills	222	34	
Vomiting	226	30	

	**M**	**SD**	

Age began losing weight for competitions (years)	20.37	6.46	El < Am (0.45) Wr < boxing (0.75), BJJ (0.81), & MT/KB (0.87)
Frequency of weight loss before a competition in the last 2 years (Likert 1–5)	3.73	1.38	S/R < Am (0.41) & El (0.53) Judo < boxing (0.89), MT/KB (0.89), & MMA (1.02)
Typical weight loss before a competition (kg)	5.16	2.90	S/R > Am (0.49) MMA > BJJ (0.92) & judo (1.29) MT/KB > BJJ (1.10) & judo (1.54)
Most weight loss before a competition (kg)	7.89	4.52	Wr > BJJ (0.76)
Typical weight loss in the 2 weeks prior to a competition (kg)	3.64	2.10	MMA > BJJ (0.64) & judo (0.87) MT/KB > BJJ (0.63), judo (0.73), & TSS (1.01) Wr > boxing (0.65), BJJ (0.91), judo (1.13), & TSS (1.20)
Typical weight loss in the 24 h prior to a competition (kg)	1.64	1.51	El > S/R (0.57) MMA > BJJ (0.62), boxing (0.66), TSS (0.87), & judo (0.90) MT/KB > boxing (0.77), TSS (1.00), & judo (1.07) Wr > boxing (0.71) & TSS (0.93)
Weight gain after weigh-in for a competition (kg)	2.47	2.11	MT/KB > Wr (0.68), boxing (0.80), BJJ (0.85), TSS (1.03), & judo (1.67) MMA > boxing (0.72), BJJ (0.77), TSS (0.93), & judo (1.47)

*Note:* All findings appear in Appendices B–D.

Abbreviations: Am = amateurs, BJJ = Brazilian jiu-jitsu, El = elite, MMA = mixed martial arts, MT/KB = muay thai/kick boxing, S/R = state or regional, TSS = traditional striking sports, Wr = wrestling.

## Data Availability

The data that support the findings of this study are available from the corresponding author upon reasonable request.

## Supporting Information

Appendix A describes the initial screening of the data and provides context on outlier data. Supporting files. The remaining appendices provide additional nonsignificant results that could not be included in the main manuscript.
