# Supplementary material for: Rapid Weight Loss Across Combat Sports and the Relationships Between Methods and Magnitude
Source: Transl Sports Med. 2025 Mar 26;2025:2946317. doi: 10.1155/tsm2/2946317 (PMC11964705; doi:10.1155/tsm2/2946317)
Supplement: Supporting Information — Additional supporting information can be found online in the Supporting Information section. [file 2946317.f1.docx]

**Appendix A**

**Details of the statistical analyses**

Preliminary analysis for pre-competition weight loss history and common methods or pre-competition RWL involved examining the data for univariate outliers (z > 3.29 – see Tabachnick et al. (2013).

For one-way ANOVA, descriptive statistics (*M* & *SD*) were reported. If the findings of the one-way ANOVA were statistically significant between the groups (*p* < 05), Tukey’s post-hoc test used to examine how the two groups were different on the descriptive data. Cohen’s criteria (Cohen, 2013) of .20 (small), .50 (medium), and .80 (large) were used to describe effect size (*d*).

For analysis according Pearson *r*, Cohen’s criteria (Cohen, 1988) of .10 (small), .30 (medium), and .50 (large) were used to describe effect size (*d)*. Analysis for primary combat sport, due to the small number of users spread across six groups of CS.

For χ^2^ test for independence, analysis for primary combat sport, due to the small number of users spread across six groups of CS. Inspection of the adjusted residuals was used as a form of post-hoc test, with values greater than 2 indicating that the cases were greater than expected (a positive value) or less than expected (a negative value). As suggested by Gravetter and Wallnau (2013) regarding effect size for a 2 (usage: non-users or users) x 3 (athlete: amateur, state/regional, or elite) table, Cramer’s V of .07 was defined as a small effect, a value of .21 was defined as a medium effect, and a value of .35 was defined as a large effect.

Twelve univariate outliers for history of RWL before a competition were noted. These athletes were:

- A 44-year-old regional / state female boxer from Belgium (and near the mid-point of their anticipated career) [case # 130] reported being 42 years old when they began RWL before competing. They reported never using any of the less-common methods of RWL before competing.
- A 64-year-old elite male wrestler from the USA (and near the end of their anticipated career) [case # 271] reported being 59 years old when they began RWL before competing. They reported a history of using one less-common methods of RWL (diuretics) before competing.
- A 37-year-old male amateur TSS athlete from the USA (and very close to the end of their anticipated career) [case #110] reported typically losing 20 kg before competing. They reported a history of using two less-common methods of RWL (diuretics and diet pills) before competing.
- A 31-year-old male amateur boxer from the USA (and mid-way through their anticipated career) [case # 69] reported losing 25 kg as the most weight lost before competing. They reported never using any of the less-common methods of RWL before competing.
- A 18 year old male amateur boxer from Singapore (and the beginning of their anticipated career) [case #50] reported losing 25.9 kg as the most weight lost before competing. They reported never using any of the less-common methods of RWL before competing.
- A 25-year-old Australian male amateur MMA athlete [id # 9] (and about a quarter of the way through their anticipated career) reported typically losing 12 kg two weeks before competing. The same athlete reported typically losing 8 kg in the 24 hours before weigh-in as well as typically regaining 10 kg after weigh-in and before competing. They reported a history of using four less-common methods of RWL (laxatives, diuretics, diet pills, and vomiting) before competing.
- A 25-year-old-amateur male muay thai / kick boxing athlete from the USA (near the beginning of their anticipated career) [case # 29] reported typically losing 6.80 kg in the 24 hours before weigh-in. They reported never using any of the less-common methods of RWL before competing.
- A 24-year-old male elite MMA athlete from Australia (near the beginning of their anticipated career) [case # 215] reporting typically losing 7 kg in the 24 hours before weigh-in. They reported a history of using one of the less-common methods of RWL (spitting) before competing.
- A 26-year-old male elite MMA athlete from Australia (close to the mid-point of their anticipated career) [case # 219] reported typically losing 7 kg in the 24 hours 24 hours before weigh-in. They reported a history of using six of the less common methods of RWL (winter or plastic suits for the whole day without exercising, spitting, laxatives, diuretics, diet pills, and vomiting) before competing.
- A 19-year-old male regional / state wrestler from the USA (near the end of their anticipated career [case # 179] reported typically losing 7.2 kg in the 24 hours before weigh-in. They reported a history of using three of the less common RWL (winter or plastic suits for the whole day without exercising, spitting, and vomiting) before competing.
- A 27-year-old Australian male elite MMA athlete (and near the mid-point of their anticipated career) [case # 223] reported typically regaining 10 kg after weigh-in and before competing. They reported a history of using three less-common methods of RWL (winter or plastic suits for the whole day without exercising, spitting, and laxatives).
- A 26-year-old Australian male amateur MMA athlete (and near the beginning of their anticipated career) [case # 39] reported typically regaining 10 kg after weigh-in and before competing. They reported never using any of the less-common methods of RWL before competing.

**Appendix B**

**Descriptives statistics for common methods of RWL before a competition; and all differences for these measures according to level of competition and primary combat sport**

| Typical usage of…. | Overall | |  | By group | | | | |
| --- | --- | --- | --- | --- | --- | --- | --- | --- |
|  | M | SD | *F* (*df*) *p* | Group | *n* | *M* | *SD* | (*d*) |
| Gradual dieting (Likert 1-4) | 3.53 | 0.80 | 4.16 (2, 1.47.84*) .02 | Am | 96 | 3.69 | .62 | Am > El = 0.43 |
|  |  |  |  | S/R | 88 | 3.50 | .87 |  |
|  |  |  |  | El | 72 | 3.36 | .88 |  |
|  |  |  | 1.42 (6, 94.93*) .22 | MMA | 27 | 3.63 | 0.57 | na |
|  |  |  |  | MT/KB | 41 | 3.71 | 0.56 |  |
|  |  |  |  | Boxing | 53 | 3.64 | 0.71 |  |
|  |  |  |  | BJJ | 51 | 3.51 | 0.78 |  |
|  |  |  |  | Wrestling | 36 | 3.19 | 1.04 |  |
|  |  |  |  | Judo | 22 | 3.41 | 1.01 |  |
|  |  |  |  | TSS | 26 | 3.53 | 0.80 |  |
| More exercise than usual (Likert 1-4) | 3.49 | 0.86 | 0.43 (2, 253) .64 | Am | 96 | 3.46 | 0.88 | na |
|  |  |  |  | S/R | 88 | 3.56 | 0.83 |  |
|  |  |  |  | El | 72 | 3.44 | 0.87 |  |
|  |  |  | 2.34 (6, 90.17*) .04 | MMA | 27 | 3.19 | 0.96 | na |
|  |  |  |  | MT/KB | 41 | 3.71 | 0.72 |  |
|  |  |  |  | Boxing | 53 | 3.36 | 0.96 |  |
|  |  |  |  | BJJ | 51 | 3.49 | 0.81 |  |
|  |  |  |  | Wrestling | 36 | 3.76 | 0.55 |  |
|  |  |  |  | Judo | 22 | 3.55 | 0.96 |  |
|  |  |  |  | TSS | 26 | 3.49 | 0.86 |  |
| Skipping one or two meals (Likert 1-4) | 2.84 | 1.06 | 2.67 (2, 253) .07 | Am | 96 | 2.68 | 1.04 | na |
|  |  |  |  | S/R | 88 | 3.03 | 1.01 |  |
|  |  |  |  | El | 72 | 2.82 | 1.01 |  |
|  |  |  | 8.17 (6, 94,27*) <.01 | MMA | 27 | 2.89 | 1.16 | Wr > Boxing (1.01), BJJ (1.09), TSS (1.18), & MT/KB (1.29)  Judo > MT/KB (0.70) |
|  |  |  |  | MT/KB | 41 | 2.41 | 1.12 |  |
|  |  |  |  | Boxing | 50 | 2.77 | 0.96 |  |
|  |  |  |  | BJJ | 51 | 2.73 | 0.92 |  |
|  |  |  |  | Wrestling | 36 | 3.56 | 0.65 |  |
|  |  |  |  | Judo | 22 | 3.23 | 1.23 |  |
|  |  |  |  | TSS | 26 | 2.50 | 1.06 |  |
| Fasting (Likert 1-4)0 | 2.54 | 1.23 | 0.52 (2, 253) .59 | Am | 96 | 2.48 | 1.17 | na |
|  |  |  |  | S/R | 88 | 2.51 | 1.23 |  |
|  |  |  |  | El | 72 | 2.67 | 1.30 |  |
|  |  |  | 2.71 (6, 249) .02 | MMA | 27 | 2.59 | 1.31 | Wr > Boxing (0.75) & BJJ (0.79) |
|  |  |  |  | MT/KB | 41 | 2.34 | 1.18 |  |
|  |  |  |  | Boxing | 53 | 2.34 | 1.13 |  |
|  |  |  |  | BJJ | 51 | 2.27 | 1.20 |  |
|  |  |  |  | Wrestling | 36 | 3.14 | 1.05 |  |
|  |  |  |  | Judo | 22 | 2.91 | 1.41 |  |
|  |  |  |  | TSS | 26 | 2.62 | 1.30 |  |
|  |  |  |  |  |  |  |  |  |
|  |  |  |  |  |  |  |  |  |
|  |  |  |  |  |  |  |  |  |
|  |  |  |  |  |  |  |  |  |
| Restricting fluid ingestion (Likert 1-4) | 2.83 | 1.22 | 4.16 (2, 165.44*) .03 | Am | 96 | 2.81 | 1.18 | El > S/R (0.45) |
|  |  |  |  | S/R | 88 | 2.60 | 1.34 |  |
|  |  |  |  | El | 72 | 3.14 | 1.07 |  |
|  |  |  | 5.16 (6, 95.52*) < .01 | MMA | 27 | 2.81 | 1.15 | TSS < Judo (0.79), MT/KB (1.06), & Wr (1.33). |
|  |  |  |  | MT/KB | 41 | 3.17 | 1.10 |  |
|  |  |  |  | Boxing | 53 | 2.68 | 1.21 |  |
|  |  |  |  | BJJ | 51 | 2.67 | 1.19 |  |
|  |  |  |  | Wrestling | 36 | 3.39 | 0.96 |  |
|  |  |  |  | Judo | 22 | 3.05 | 1.53 |  |
|  |  |  |  | TSS | 26 | 2.00 | 1.13 |  |
| Excessive fluid ingestion (Likert 1-4) | 3.13 | 1.29 | 3.42 (2, 161.07*) .04 | Am | 96 | 3.24 | 1.30 | na |
|  |  |  |  | S/R | 88 | 2.85 | 1.18 |  |
|  |  |  |  | El | 72 | 3.22 | 1.36 |  |
|  |  |  | 5.21 (6, 96.04*) <.01 | MMA | 27 | 3.59 | 1.42 | MT/KB > TSS (0.93), Judo (0.96), & Wr (1.08).  MMA > Wr (0.81) |
|  |  |  |  | MT/KB | 41 | 3.78 | 1.24 |  |
|  |  |  |  | Boxing | 53 | 3.13 | 1.21 |  |
|  |  |  |  | BJJ | 51 | 3.20 | 1.31 |  |
|  |  |  |  | Wrestling | 36 | 2.59 | 1.00 |  |
|  |  |  |  | Judo | 22 | 2.64 | 1.18 |  |
|  |  |  |  | TSS | 26 | 2.65 | 1.23 |  |
|  |  |  |  |  |  |  |  |  |
|  |  |  |  |  |  |  |  |  |
| Training in heated rooms (Likert 1-4) | 2.42 | 1.26 | 3.00 (2, 253) .05 | Am | 96 | 2.22 | 1.14 | na |
|  |  |  |  | S/R | 88 | 2.42 | 1.35 |  |
|  |  |  |  | El | 72 | 2.69 | 1.27 |  |
|  |  |  | 12.44 (6, 95.96*) < .01 | MMA | 27 | 2.48 | 1.16 | Wr > MMA (0.97), Boxing (1.07), MT/KB (1.19), BJJ (1.45) & TSS (1.96)  Judo > TSS (0.91) |
|  |  |  |  | MT/KB | 41 | 2.20 | 1.29 |  |
|  |  |  |  | Boxing | 53 | 2.42 | 1.18 |  |
|  |  |  |  | BJJ | 51 | 2.04 | 1.17 |  |
|  |  |  |  | Wrestling | 36 | 3.47 | 0.85 |  |
|  |  |  |  | Judo | 22 | 2.82 | 1.47 |  |
|  |  |  |  | TSS | 26 | 1.69 | 0.97 |  |
| Sauna (Likert 1-4) | 2.48 | 1.22 | 1.86 (2, 253) .16 | Am | 96 | 2.49 | 1.13 | na |
|  |  |  |  | S/R | 88 | 2.31 | 1.28 |  |
|  |  |  |  | El | 72 | 2.68 | 1.25 |  |
|  |  |  | 12.56 (6, 96.93*) <.01 | MMA | 27 | 2.96 | 1.19 | Wr > BJJ (0.80) & TSS (2.10)  TSS < BJJ (0.93), Boxing (1.19), MT/KB (1.48), MMA (1.63) & Wr (2.10) |
|  |  |  |  | MT/KB | 41 | 2.76 | 1.20 |  |
|  |  |  |  | Boxing | 53 | 2.42 | 1.12 |  |
|  |  |  |  | BJJ | 51 | 2.25 | 1.25 |  |
|  |  |  |  | Wrestling | 36 | 3.11 | 0.95 |  |
|  |  |  |  | Judo | 22 | 2.32 | 1.39 |  |
|  |  |  |  | TSS | 26 | 1.38 | 0.75 |  |

Notes:

*N*= 256. na= not applicable.* Welch test reported because the assumption of homogeneity of variance was violated. Am= Amateurs. S/R = State or Regional. El= Elite. TSS= Traditional Striking Sports; Wr= Wrestling; MMA= Mixed Martial Arts; MT/KB = Muay Thai / Kick Boxing; BJJ= Brazilian jiu-jitsu.

**Appendix C**

**Usage of less common methods of RWL; and all differences for these measures according to level of participation**

|  |  | *n* | χ^2^ (*p*) Cramer’s *V* (effect size) |  | By level |  |
| --- | --- | --- | --- | --- | --- | --- |
|  |  |  |  | Amateurs | State or Regional | Elite |
| Plastic/rubber suits or towel wrapping | Not used | 132 | 13.34 (<.01) .23 (medium) | 56 | 52 | 24* |
|  | Used | 124 |  | 40 | 36 | 48* |
| Use of winter or plastic suits for whole day (without exercising) | Not used | 187 | 10.69 (<.01) .20 (medium) | 79* | 65 | 43* |
|  | Used | 69 |  | 17* | 23 | 29* |
| Spitting | Not used | 171 | 11.49 (<.01) .21 (medium) | 76* | 55 | 40* |
|  | Used | 85 |  | 20* | 33 | 32* |
| Laxatives | Not used | 206 | 4.39 (.11) .12 | 81 | 73 | 52 |
|  | Used | 50 |  | 15 | 15 | 20 |
| Diuretics | Not used | 208 | 2.33 (.24) .11 | 79 | 75 | 55 |
|  | Used | 48 |  | 17 | 13 | 18 |
| Diet Pills | Not used | 222 | 3.69 (.16) .12 | 87 | 77 | 58 |
|  | Used | 34 |  | 9 | 11 | 14 |
| Vomiting | Not used | 226 | 3.01 (.22) .11 | 89 | 76 | 61 |
|  | Used | 30 |  | 7 | 12 | 11 |

Notes:

*N*= 256. *df=* 2 for all tests. * indicates adjusted residual <±2. Differences between type of combat sport for less common methods of RWL were not explored because of the small number of users, meaning that such analyses would have been underpowered.

**Appendix D**

**Descriptives statistics for pre-competition weight loss history; and all differences for these measures according to level of competition and primary combat sport**

|  | Overall | |  | By | group |  |  |  |
| --- | --- | --- | --- | --- | --- | --- | --- | --- |
|  | *M* | *SD* | *F* (*df*) | Group | *n* | *M* | *SD* | (*d*) |
| Age began losing weight for competitions (years) | 20.37 | 6.46 | 3.89 (2, 253) .02 | Am | 96 | 21.60 | 5.54 | Am > El (0.45) |
|  |  |  |  | S/R | 88 | 20.28 | 6.93 |  |
|  |  |  |  | El | 72 | 18.83 | 6.73 |  |
|  |  |  | 5.43 (6, 249) <.01 | MMA | 27 | 19.59 | 4.79 | Wr < Boxing (0.75), BJJ (0.81), & MT/KB (0.87) |
|  |  |  |  | MT/KB | 41 | 22.07 | 5.28 |  |
|  |  |  |  | Boxing | 53 | 21.53 | 5.94 |  |
|  |  |  |  | BJJ | 51 | 22.27 | 7.05 |  |
|  |  |  |  | Wrestling | 36 | 15.86 | 8.54 |  |
|  |  |  |  | Judo | 22 | 18.18 | 4.65 |  |
|  |  |  |  | TSS | 26 | 20.50 | 3.80 |  |
| Frequency of weight loss before a competition in the last 2 years (Likert 1-5) | 3.73 | 1.38 | 6.52 (2, 253) <.01 | Am | 96 | 3.88 | 1.32 | S/R < Am (0.41) & Elite (0.53) |
|  |  |  |  | S/R | 88 | 3.32 | 1.44 |  |
|  |  |  |  | El | 72 | 4.04 | 1.30 |  |
|  |  |  | 4.22 (6, 249) <.01 | MMA | 27 | 4.19 | 1.15 | Judo < Boxing (0.89), MT/KB (0.89), & MMA (1.02) |
|  |  |  |  | MT/KB | 41 | 4.10 | 1.34 |  |
|  |  |  |  | Boxing | 53 | 4.08 | 1.19 |  |
|  |  |  |  | BJJ | 51 | 3.43 | 1.35 |  |
|  |  |  |  | Wrestling | 36 | 3.78 | 1.44 |  |
|  |  |  |  | Judo | 22 | 2.86 | 1.46 |  |
|  |  |  |  | TSS | 26 | 3.23 | 1.51 |  |
| Typical weight loss before a competition (kg) | 5.16 | 2.90 | 5.35 (2, 253) <.01 | Am | 96 | 5.85 | 3.15 | S/R > Am (0.49) |
|  |  |  |  | S/R | 88 | 4.47 | 2.49 |  |
|  |  |  |  | El | 72 | 5.09 | 2.86 |  |
|  |  |  | 6.61 (6,249) <.01 | MMA | 27 | 6.44 | 2.94 | MMA > BJJ (0.92) & Judo (1.29)  MT/KB > BJJ (1.10) & Judo (1.54) |
|  |  |  |  | MT/KB | 41 | 6.78 | 2.80 |  |
|  |  |  |  | Boxing | 53 | 5.29 | 2.81 |  |
|  |  |  |  | BJJ | 51 | 3.95 | 2.31 |  |
|  |  |  |  | Wrestling | 36 | 5.17 | 2.27 |  |
|  |  |  |  | Judo | 22 | 3.41 | 1.85 |  |
|  |  |  |  | TSS | 26 | 4.86 | 3.91 |  |
| Most weight loss before a competition (kg) | 7.89 | 4.52 | 3.48 (2, 253) .03 | Am | 96 | 8.38 | 5.11 | na |
|  |  |  |  | S/R | 88 | 6.88 | 4.05 |  |
|  |  |  |  | El | 72 | 8.49 | 4.05 |  |
|  |  |  | 4.56 (6, 96.65*) <.01 | MMA | 27 | 8.70 | 3.88 | Wr > BJJ (0.76) |
|  |  |  |  | MT/KB | 41 | 8.80 | 4.08 |  |
|  |  |  |  | Boxing | 53 | 8.51 | 6.00 |  |
|  |  |  |  | BJJ | 51 | 6.21 | 3.15 |  |
|  |  |  |  | Wrestling | 36 | 9.16 | 4.40 |  |
|  |  |  |  | Judo | 22 | 5.80 | 3.16 |  |
|  |  |  |  | TSS | 26 | 7.69 | 4.56 |  |
|  |  |  |  |  |  |  |  |  |
|  |  |  |  |  |  |  |  |  |
|  |  |  |  |  |  |  |  |  |
| Typical weight loss in the two weeks prior to a competition (kg) | 3.64 | 2.10 | 2.44 (2, 157.99*) .19 | Am | 96 | 3.66 | 2.01 | na |
|  |  |  |  | S/R | 88 | 3.29 | 1.90 |  |
|  |  |  |  | El | 72 | 4.04 | 2.40 |  |
|  |  |  | 6.89 (6, 249) < .01 | MMA | 27 | 4.46 | 2.57 | MMA > BJJ (0.64)& Judo (0.87)  MT/KB > BJJ (0.63), Judo (0.73) & TSS (1.01)  Wr > Boxing (0.65), BJJ (0.91), Judo (1.13) & TSS (1.20) |
|  |  |  |  | MT/KB | 41 | 4.47 | 2.31 |  |
|  |  |  |  | Boxing | 53 | 3.41 | 1.93 |  |
|  |  |  |  | BJJ | 51 | 3.02 | 1.54 |  |
|  |  |  |  | Wrestling | 36 | 4.68 | 2.02 |  |
|  |  |  |  | Judo | 22 | 2.65 | 1.69 |  |
|  |  |  |  | TSS | 26 | 2.55 | 1.64 |  |
| Typical weight loss in the 24 hours prior to a competition (kg) | 1.64 | 1.51 | 6.92 (2, 253) < .01 | Am | 96 | 1.59 | 1.48 | El > S/R (0.57) |
|  |  |  |  | S/R | 88 | 1.29 | 1.31 |  |
|  |  |  |  | El | 72 | 2.15 | 1.65 |  |
|  |  |  | 6.09 (6, 96.21) <.01 | MMA | 27 | 2.48 | 2.20 | MMA > BJJ (0.62), Boxing (0.66), TSS (0.87), & Judo (0.90)  MT/KB > Boxing (0.77), TSS (1.00), & Judo (1.07)  Wr > Boxing (0.71) & TSS (0.93) |
|  |  |  |  | MT/KB | 41 | 2.34 | 1.65 |  |
|  |  |  |  | Boxing | 53 | 1.24 | 1.13 |  |
|  |  |  |  | BJJ | 51 | 1.30 | 1.22 |  |
|  |  |  |  | Wrestling | 36 | 2.16 | 1.41 |  |
|  |  |  |  | Judo | 22 | 1.04 | 0.94 |  |
|  |  |  |  | TSS | 26 | 0.98 | 1.19 |  |
|  |  |  |  |  |  |  |  |  |
|  |  |  |  |  |  |  |  |  |
|  |  |  |  |  |  |  |  |  |
| Weight gain after weigh-in for a competition (kg) | 2.47 | 2.11 | 6.50 (2, 156.23*) <.01 | Am | 96 | 2.54 | 2.09 | El > S/R (0.55) |
|  |  |  |  | S/R | 88 | 1.90 | 1.70 |  |
|  |  |  |  | El | 72 | 3.07 | 2.43 |  |
|  |  |  | 9.20 (6, 98.97*) < .01 | MMA | 27 | 3.75 | 2.52 | MT/KB > Wr (0.68), Boxing (0.80), BJJ (0.85), TSS (1.03), & Judo (1.67)  MMA > Boxing (0.72), BJJ (0.77), TSS (0.93), & Judo (1.47) |
|  |  |  |  | MT/KB | 41 | 3.89 | 2.54 |  |
|  |  |  |  | Boxing | 53 | 2.14 | 1.66 |  |
|  |  |  |  | BJJ | 51 | 1.97 | 1.93 |  |
|  |  |  |  | Wrestling | 36 | 2.46 | 1.66 |  |
|  |  |  |  | Judo | 22 | 1.12 | 0.97 |  |
|  |  |  |  | TSS | 26 | 1.78 | 1.74 |  |

Notes:

*N*= 256. * Welch test reported because the assumption of homogeneity of variance was violated. Am= Amateurs. S/R = State or Regional. El= Elite. TSS= Traditional Striking Sports; Wr= Wrestling; MMA= Mixed Martial Arts; MT/KB = Muay Thai / Kick Boxing. BJJ= Brazilian jiu-jitsu.

**Appendix E**

**All correlations between pre-competition weight loss history with common methods of RWL before a competition.**

| Typical usage of…. | Age began losing weight for competitions  (years) | Frequency of weight loss before a competition in the last 2 years (Likert 1-5) | Typical weight loss before a competition (kg) | Most weight loss before a competition (kg) | Typical weight loss in the two weeks prior to a competition (kg) | Typical weight loss in the 24 hours prior to a competition (kg) | Weight gain after weigh-in for a competition (kg) |
| --- | --- | --- | --- | --- | --- | --- | --- |
| Gradual dieting (Likert 1-4) | .13* | .07 | .16* | .05 | -.06 | -.03 | .04 |
| More exercise than usual (Likert 1-4) | -.14* | .05 | .18* | .16* | .16* | .03 | .07 |
| Skipping one or two meals (Likert 1-4) | -.14* | .08 | .04 | .14* | .19* | .02 | .05 |
| Fasting (Likert 1-4) | -.20* | .06 | .12* | .23* | .21* | .14* | 17* |
| Restricting fluid ingestion (Likert 1-4) | -.25* | .19* | .18* | .22* | .38* | .40* | .28* |
| Excessive fluid ingestion (Likert 1-4) | .02 | .25* | .29* | .15* | .36* | .47* | .45* |
| Training in heated rooms (Likert 1-4) | -.28* | .05 | .19* | .24* | .28* | .23* | .15* |
| Sauna (Likert 1-4) | -.08 | .13* | .29* | .22* | .38* | .45* | .41* |

Notes:

*N*= 256. * indicates *p*< .05.

**Appendix F**

**Findings for the multigroup analyses for Table 4**

|  | Level  (*df*= 2) |
| --- | --- |
| Gradual dieting |  |
| Age began losing weight for competitions | χ^2^= 2.23, *p*= .33 |
| Frequency of weight loss before a competition in the last 2 years | χ^2^= 0.11, *p*= .95 |
| Typical weight loss before a competition | χ^2^= 1.72, *p*= .43 |
| Most weight loss before a competition | χ^2^=2.52, *p*= .28 |
| Typical weight loss in the two weeks prior to a competition | χ^2^= 2.62, *p*= .26 |
| Typical weight loss in the 24 hours prior to a competition | χ^2^= 4.56, *p*= .10 |
| Weight gain after weigh-in for a competition | χ^2^= 3.74, *p*= .15 |
| More exercise than usual |  |
| Age began losing weight for competitions | χ^2^= 1.68, *p*= .43 |
| Frequency of weight loss before a competition in the last 2 years | χ^2^= 1.06, *p*= .59 |
| Typical weight loss before a competition | χ^2^= 0.54, *p*< .77 |
| Most weight loss before a competition | χ^2^= 3.17, *p*= .21 |
| Typical weight loss in the two weeks prior to a competition | χ^2^= 0.37, *p*= .83 |
| Typical weight loss in the 24 hours prior to a competition | χ^2^= 1.01, *p*= .60 |
| Weight gain after weigh-in for a competition | χ^2^= 1.01, *p*= .60 |
| Skipping one or two meals |  |
| Age began losing weight for competitions | χ^2^= 0.02, *p*= .99 |
| Frequency of weight loss before a competition in the last 2 years | χ^2^= 0.01, *p*= .99 |
| Typical weight loss before a competition | χ^2^= 1.23, *p*= .54 |
| Most weight loss before a competition | χ^2^= 0.96, *p*= .63 |
| Typical weight loss in the two weeks prior to a competition | χ^2^= 0.07, *p*= .96 |
| Typical weight loss in the 24 hours prior to a competition | χ^2^= 0.07, *p*= .97 |
| Weight gain after weigh-in for a competition | χ^2^= 0.45, *p*= .78 |
| Fasting |  |
| Age began losing weight for competitions | χ^2^= 3.60, *p*= .17 |
| Frequency of weight loss before a competition in the last 2 years | χ^2^= 1.56, *p*= .47 |
| Typical weight loss before a competition | χ^2^= 0.04, *p*= .98 |
| Most weight loss before a competition | χ^2^= 0.16, *p*= .93 |
| Typical weight loss in the two weeks prior to a competition | χ^2^= 2.99, *p*= .22 |
| Typical weight loss in the 24 hours prior to a competition | χ^2^= 3.64, *p*= .16 |
| Weight gain after weigh-in for a competition | χ^2^= 2.09, *p*= .35 |
|  |  |
| Restricting fluid ingestion |  |
| Age began losing weight for competitions | χ^2^= 9.50, *p <*.01 |
| Frequency of weight loss before a competition in the last 2 years | χ^2^= 1.59, *p*= .45 |
| Typical weight loss before a competition | χ^2^= 1.44, *p*= .49 |
| Most weight loss before a competition | χ^2^= 0.81, *p*= .67 |
| Typical weight loss in the two weeks prior to a competition | χ^2^= 1,56, *p*= .47 |
| Typical weight loss in the 24 hours prior to a competition | χ^2^= 0.82, *p*= .66 |
| Weight gain after weigh-in for a competition | χ^2^= 0.12, *p*= .94 |
| Excessive fluid ingestion |  |
| Age began losing weight for competitions | χ^2^= 0.77, *p*= .68 |
| Frequency of weight loss before a competition in the last 2 years | χ^2^= 7.83, *p*= .02 |
| Typical weight loss before a competition | χ^2^= 4.07, *p*= .13 |
| Most weight loss before a competition | χ^2^= 1.25, *p*= .53 |
| Typical weight loss in the two weeks prior to a competition | χ^2^= 5.03, *p*= .08 |
| Typical weight loss in the 24 hours prior to a competition | χ^2^= 2.95, *p*= .23 |
| Weight gain after weigh-in for a competition | χ^2^= 3.38, *p*= .19 |
| Training in heated rooms |  |
| Age began losing weight for competitions | χ^2^= 6.05, *p*= .05* |
| Frequency of weight loss before a competition in the last 2 years | χ^2^= 5.92, *p*= .02** |
| Typical weight loss before a competition | χ^2^= 0.20, *p*= .91 |
| Most weight loss before a competition | χ^2^= 0.76, *p*= .69 |
| Typical weight loss in the two weeks prior to a competition | χ^2^= 1.34, *p*= .51 |
| Typical weight loss in the 24 hours prior to a competition | χ^2^= 1.62, *p*= .44 |
| Weight gain after weigh-in for a competition | χ^2^= 0.08, *p*= .96 |
| Sauna |  |
| Age began losing weight for competitions | χ^2^= 1.60, *p*= .45 |
| Frequency of weight loss before a competition in the last 2 years | χ^2^= 3.21, *p*= .20 |
| Typical weight loss before a competition | χ^2^= 1.35, *p*= .51 |
| Most weight loss before a competition | χ^2^= 1.55, *p*= .46 |
| Typical weight loss in the two weeks prior to a competition | χ^2^= 2.52, *p*= .28 |
| Typical weight loss in the 24 hours prior to a competition | χ^2^= 0.96, *p*= .62 |
| Weight gain after weigh-in for a competition | χ^2^= 0.12, *p*= .94 |

Note

* was <.05. ** was > .05

**Appendix G**

**All differences for pre-competition weight loss history according to the use or non-use of less common methods of weight loss before a competition: Age began losing weight for competitions; frequency of weight loss before a competition in the last 2 years; and typical weight loss before a competition**

|  | Age began losing weight for competitions (years) | | | | | | Frequency of weight loss before a competition in the last 2 years  (Likert 1-5) | | | | | Typical weight loss before a competition  (kg) | | | | |
| --- | --- | --- | --- | --- | --- | --- | --- | --- | --- | --- | --- | --- | --- | --- | --- | --- |
|  |  | *F (df) p* | *n* | *M* | *SD* | *d* | *F (df) p* | *n* | *M* | *SD* | *d* | *F (df) p* | *n* | *M* | *SD* | *d* |
| Plastic/rubber suits or towel wrapping | Not used | 17.25  (1, 239.91*)  < .01 | 132 | 21.93 | 7.11 | 0.52 | 18.83  (1, 254)  < .01 | 132 | 3.38 | 1.37 | 0.54 | 20.41  (1,254)  < .01 | 132 | 4.39 | 2.87 | 0.57 |
|  | Used |  | 124 | 18.71 | 5.21 |  |  | 124 | 4.10 | 1.30 |  |  | 124 | 5.98 | 2.72 |  |
| Use of winter or plastic suits for whole day (without exercising) | Not used | 12.19  (1, 253.99)  < .01 | 187 | 21.21 | 6.60 | 0.54 | 2.89  (1, 254)  .09 | 187 | 3.64 | 1.41 | na | 5.18  (1,254)  .02 | 187 | 4.91 | 2.91 | 0.33 |
|  | Used |  | 69 | 18.14 | 5.34 |  |  | 69 | 3.97 | 1.28 |  |  | 69 | 5.83 | 2.79 |  |
| Spitting | Not used | 44.38  (1, 254)  <.01 | 171 | 22.12 | 6.29 | 0.95 | 2.77  (1, 178.40)  .10 | 171 | 3.63 | 1.40 | na | 6.54  (1,254)  .01 | 171 | 4.84 | 2.93 | 0.35 |
|  | Used |  | 85 | 16.85 | 5.25 |  |  | 85 | 3.73 | 1.38 |  |  | 85 | 5.81 | 2.75 |  |
| Laxatives | Not used | 2.05  1, 254)  .15 | 206 | 20.66 | 6.31 | na | 2.74  (1, 254)  .99 | 206 | 3.66 | 1.34 | na | 9.05  (1, 254)  <.01 | 206 | 4.89 | 2.82 | 0.47 |
|  | Used |  | 50 | 19.20 | 6.98 |  |  | 50 | 4.02 | 1.39 |  |  | 50 | 6.28 | 2.99 |  |
| Diuretics | Not used | 1.03  (1, 254)  .31 | 208 | 20.57 | 5.99 | na | 4.36  (1, 254)  .04 | 208 | 3.64 | 1.39 | 0.35 | 1.48  (1, 254)  <.01 | 208 | 4.78 | 2.71 | 0.48 |
|  | Used |  | 48 | 19.20 | 8.19 |  |  | 48 | 4.10 | 1.31 |  |  | 48 | 6.36 | 3.39 |  |
| Diet Pills | Not used | 13.24  (1,254)  < .01 | 222 | 20.93 | 6.47 | 0.80 | 2.22  (1. 254)  .40 | 222 | 3.68 | 1.38 | na | 7.05  (1, 254)  <.01 | 222 | 4.97 | 2.71 | 0.40 |
|  | Used |  | 34 | 16.71 | 5.11 |  |  | 34 | 4.06 | 1.39 |  |  | 34 | 6.41 | 3.72 |  |
| Vomiting | Not used | 35.97  (1, 48,25*)  < .01 | 226 | 21.00 | 6.44 | 1.17 | 1.63  (1, 254)  .20 | 218 | 3.70 | 1.37 | na | 1.74  (1, 254)  .19 | 226 | 5.08 | 2.94 | na |
|  | Used |  | 30 | 15.63 | 4.30 |  |  | 27 | 4.03 | 1.47 |  |  | 30 | 5.82 | 1.52 |  |

Notes:

* Welch test reported because the assumption of homogeneity of variance was violated. na= not applicable.

**Appendix H**

**All differences for pre-competition weight loss history according to the use or non-use of less common methods of weight loss before a competition: Most weight loss before a competition; and typical weight loss in the two weeks prior to a competition**

|  | Most weight loss before a competition  (kg) | | | | | | Typical weight loss in the two weeks prior to a competition  (kg) | | | | |
| --- | --- | --- | --- | --- | --- | --- | --- | --- | --- | --- | --- |
|  |  | *F (df) p* | *n* | *M* | *SD* | *d* | *F (df) p* | *n* | *M* | *SD* | *d* |
| Plastic/rubber suits or towel wrapping | Not used | 18.50  (1, 254)  <.01 | 132 | 6.75 | 4.43 | 0.54 | 52,90  (1, 254)  <.01 | 132 | 2.80 | 1.66 | 0.90 |
|  | Used |  | 124 | 9.11 | 4.30 |  |  | 124 | 4.54 | 2.16 |  |
| Use of winter or plastic suits for whole day (without exercising) | Not used | 18.50  (1, 254) <.01 | 187 | 7.26 | 4.21 | 0.50 | 18.84  (1,243)  <.01 | 187 | 3.32 | 1.99 | 0.56 |
|  | Used |  | 69 | 9.61 | 4.90 |  |  | 69 | 4.51 | 2.17 |  |
| Spitting | Not used | 16.00  (1,254) <.01 | 171 | 7.12 | 4.45 | 0.54 | 31.08  (1, 140.78)  <.01 | 171 | 3.12 | 1.82 | 0.74 |
|  | Used |  | 85 | 9.45 | 4.27 |  |  | 85 | 4.68 | 2.25 |  |
| Laxatives | Not used | 19.28  (1,254) <.01 | 206 | 7.35 | 4.27 | 0.59 | 11.98  (1,64.51)  <.01 | 206 | 3.39 | 1.93 | 0.54 |
|  | Used |  | 50 | 10.14 | 4.84 |  |  | 50 | 4.67 | 2.45 |  |
| Diuretics | Not used | 9.47  (1, 254)  <.01 | 208 | 7.48 | 4.36 | 0.47 | 11.04  (1, 254)  <.01 | 208 | 3.43 | 2.00 | 0.49 |
|  | Used |  | 48 | 9.68 | 4.81 |  |  | 48 | 4.53 | 2.30 |  |
| Diet Pills | Not used | 13.32  (1 ,254)  <.01 | 222 | 7.50 | 4.40 | 0.66 | 12.58  (1, 254)  <.01 | 222 | 3.46 | 1.98 | 0.65 |
|  | Used |  | 34 | 10.47 | 4.51 |  |  | 34 | 4.81 | 2.10 |  |
| Vomiting | Not used | 8.61  (1, 254)  <.01 | 226 | 7.60 | 4.54 | 0.66 | 13.82  (1, 243)  <.01 | 226 | 3.45 | 1.98 | 0.61 |
|  | Used |  | 30 | 10.14 | 3.74 |  |  | 30 | 4.94 | 2.52 |  |

Notes:

* Welch test reported because the assumption of homogeneity of variance was violated.

**Appendix J**

**All differences for pre-competition weight loss history according to the use or non-use of less common methods of weight loss before a competition: Typical weight loss in the 24 hours prior to a competition; and weight gain after weigh-in for a competition**

|  | Typical weight loss in the 24 hours prior to a competition  (kg) | | | | | | Weight gain after weigh-in for a competition  (kg) | | | | |
| --- | --- | --- | --- | --- | --- | --- | --- | --- | --- | --- | --- |
|  |  | *F (df) p* | *n* | *M* | *SD* | *d* | *F (df) p* | *n* | *M* | *SD* | *d* |
| Plastic/rubber suits or towel wrapping | Not used | 45.84  (1, 211.19*)  <.01 | 132 | 1.07 | 1.09 | 0.85 | 28.39  (1, 225.04*)  <.01 | 132 | 1.80 | 1.68 | 0.66 |
|  | Used |  | 124 | 2.26 | 1.65 |  |  | 124 | 3.12 | 2.29 |  |
| Use of winter or plastic suits for whole day (without exercising) | Not used | 11.31  (1 ,254)  <.01 | 187 | 1.46 | 1.43 | 0.45 | 4.23  (1 ,254)  .04 | 187 | 2.31 | 2.09 | 0.29 |
|  | Used |  | 69 | 2.16 | 1.61 |  |  | 69 | 2.92 | 2.14 |  |
| Spitting | Not used | 22.51  (1, 254)  <.01 | 171 | 1.34 | 1.38 | 0.60 | 12.01  (1, 254)  <.01 | 171 | 2.16 | 2.26 | 0.45 |
|  | Used |  | 85 | 2.25 | 1.58 |  |  | 85 | 3.11 | 2.02 |  |
| Laxatives | Not used | 10.57  (1, 254)  <.01 | 206 | 1.50 | 1.41 | 0.45 | 7.74  (1, 63.26)  <.01 | 206 | 2.26 | 1.94 | 0.42 |
|  | Used |  | 50 | 2.26 | 1.74 |  |  | 50 | 3.30 | 2.57 |  |
| Diuretics | Not used | 7.54  (1, 254)  <.01 | 208 | 1.52 | 1.45 | 0.41 | 5.17  (1, 61.17*)  .03 | 208 | 2.31 | 1.98 | 0.36 |
|  | Used |  | 48 | 2.18 | 1.63 |  |  | 48 | 3.19 | 2.51 |  |
| Diet Pills | Not used | 4.37  (1, 38.90*)  .04 | 222 | 1.55 | 1.42 | 0.38 | 3.92  (1, 39.21*)  .06 | 222 | 2.35 | 2.00 | na |
|  | Used |  | 34 | 2.25 | 1.89 |  |  | 34 | 3.27 | 2.61 |  |
| Vomiting | Not used | 6.35  (1, 32,32*)  .2 | 226 | 1.53 | 1.37 | 0.49 | 4.23  (1, 33.32*)  .15 | 226 | 2.35 | 2.00 | .40 |
|  | Used |  | 30 | 2.52 | 2.11 |  |  | 30 | 3.40 | 2.71 |  |

Notes:

* Welch test reported because the assumption of homogeneity of variance was violated. na= not applicable.
